# Effect of Er: YAG laser and different surface treatment methods on the push-out bond strength of glass fiber post to self adhesive resin cement

**DOI:** 10.1007/s10103-025-04317-x

**Published:** 2025-02-08

**Authors:** Nouran Samy Mohammed Elalfy, Reham Said Elbasty

**Affiliations:** 1 Menofia University, Menofia, Egypt; 2 Cairo University, Cairo, Egypt

**Keywords:** Fiber posts, Er: YAG laser, Sandblasting, Push out test, Surface treatment

## Abstract

To compare the push-out bond strength of adhesive resin cement and glass fiber posts (GFP) at different root levels after exposure to Er: YAG laser irradiation compared to other conventional surface treatment procedures. A total of 24 mandibular premolars were decoronated, root canal treatment was done, post spaces were prepared, and roots were mounted in acrylic resin blocks. Fiber posts were divided into four groups (*n* = 6) according to surface treatment methods: (1) silane only (control group), (2) Er: YAG laser 1.5 W + silane, (3) 30% hydrogen peroxide + silane, (4) sandblasting with 50 μm aluminum oxide particles + silane. GFP were cemented using self-adhesive resin cement. Scanning electron microscope images with 500x magnification were taken for all groups. Push-out test was performed using a universal testing machine at different root levels. The difference between groups was statistically significant with laser group recording the highest mean ± SD value of push-out bond strength (5.668042 ± 1.16 MPa), followed by the H2O2 group, then the control group, meanwhile the lowest value was recorded with Sand-blasting group. There were no statistically significant differences between the Control group and Er: YAG group; Control group and sandblasted group. The difference between the radicular regions was not statistically significant, with the middle region recorded the highest push-out bond strength (4.746851 ± 0.73 MPa). GPF surface treatment using an Er: YAG laser is effective as it increases the retention to resin cement, while sandblasting decreases fiber post retention to resin cement. The hydrogen peroxide and the control groups give similar bond strength. The middle and apical regions of GFP have better retention to resin cement than the coronal one.

## Introduction

Restoring teeth that have received endodontic treatment is difficult due to the loss of structural integrity caused by caries and fractures [1, 2]. To achieve sufficient retention of the core foundation, it may be necessary to place a post into the root canal to facilitate the successful restoration of these teeth [3]. Fiber posts were developed as a response to the problems accompanied by metal posts [4]. The fibred post is composed of unidirectional fibers that are incorporated into a resin matrix [5]. Glass fiber posts contain e-glass fibers (electric glass) that consist of SiO 2, CaO, B 2O, Al 2O, and a few other oxides of alkali metals in the amorphous phase [6]. The use of these fiber posts facilitates the creation of a mechanically homogeneous monoblock, thereby diminishing the risk of fracture, as the modulus of elasticity of fiber posts is comparable to that of dentin [7]. 

The fiber post retention in the root canal depends on the bond strength between different parts (post-cement-dentin) assembly [8]. The main cause of failures in fiber posts is the interface between the fiber post and resin cement [9]. The organic component of fiber posts comprises epoxy resin, characterized by a high degree of conversion and strong cross-linking [10, 11]. As a result, the resin cement cannot completely infiltrate the surface of prefabricated fiber posts, therefore obstructing the interdiffusion process between the resin cement and resin matrix. Consequently, this polymer matrix cannot interact with the monomers of resin cement [12]. An important factor in the long-term success of restorative structures and teeth that have undergone endodontic treatment is the quality of the bonding, in which good bonding improves the distribution of stress induced by occlusal stresses [13]. Thus, in order to optimize the adhesion of resin to fiber posts, many surface pre-treatment techniques, such as mechanical or chemical treatments of the post surface, have been studied [14]. 

Surface conditioning with a silane-based resin, like 3- methacryloxypropyltrimethoxysilane, is the standard procedure [15]. Chemical bonding occurs between both the inorganic fibers and the organic components of resin-matrix cements [16]. Before this, the surface roughness can be enhanced through etching using reactive agents such as hydrofluoric acid, hydrogen peroxide, hydrochloric acid, and potassium permanganate [17]. Another option is to use either abrasive alumina or silica particles to sandblast the surface, which will increase the roughness and accordingly improve the wettability and mechanical interlocking of resin-matrix cement [18]. 

Surface treatment using novel laser-assisted methods has been investigated with respect to the laser type (e.g., Er: YAG, Nd: YAG, ErCr: YSGG laser, diode laser) and irradiation parameters (radiation levels, exposure duration, operational mode) [19–23]. Depending on the precise laser parameters, laser texturing can modify surfaces by thermal mechanical ablation. The morphological characteristics and adherence of GFP surfaces to resin-matrix cement can be improved by combining laser texturing with conventional physicochemical techniques [17]. Nonetheless, the actual effects of laser treatment and its precise parameters remain a subject of debate and require further investigation. This research was conducted to evaluate the effect of Er: YAG laser irradiation and various conventional surface treatment techniques on the push-out bond strength of GFP luted to self-adhesive resin cement at various root levels.

**Fig. 1:**
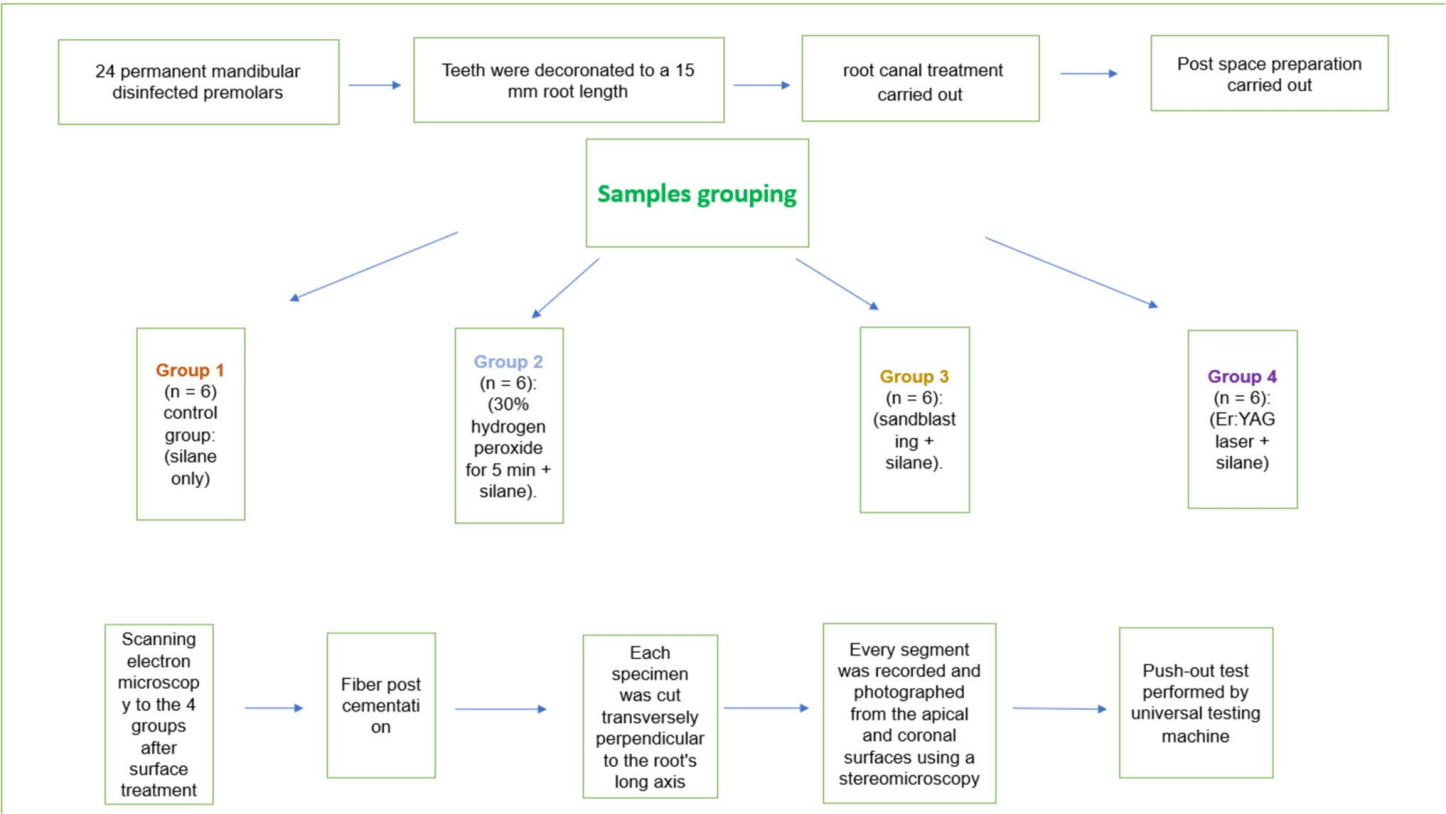
Schematic diagram of the methodology of the study

The null hypothesis of the study was that there would be no differences in the push-out bond strength when considering both GFP and resin cement after application of Er: YAG laser irradiation surface treatment, as compared to other conventional surface treatment methods (Fig. 1).

## Materials and methods

### Sample size calculation

A Cohen’s d effect size of 2 was determined using the values for push out bond strength in the cervical one third for the Er: YAG and control groups, which were derived from the results of Gomes et al. [24]. The minimum number of samples required to identify a significant difference for push-out bond strength between any two study groups was established at 24 samples (6 samples per group) when a type I error of 0.05 and a study power of 0.8 were assumed. G Power, version 3.1.9.7, was used to compute the sample size.

### Teeth selection

Twenty-four recently extracted human permanent mandibular premolars, exhibiting predominantly uniform root lengths and normal morphology, were selected from several government hospitals and health centers with periodontal and maxillofacial surgery departments. Mandibular premolars were selected based on periapical radiography and visual inspection. The criteria for sample selection included single straight-rooted mandibular premolars devoid of root caries or restorations, no previous endodontic treatments, and completely developed roots with mature apices [25]. 

### Sample preparation

Disinfection was achieved by immersing specimens in a 0.5% chloramine-T solution for 48 h. To achieve a root length of 15 mm, the teeth were decoronated using a diamond saw parallel to the cement-enamel junction under water spray. This procedure resulted in a flat surface-oriented perpendicular to the root’s longitudinal axis [25]. 

### Root canal treatment

The root canal treatment was carried out following the recommendations specified in a previous study [26]. The pulp tissues were removed using a barbed broach file barbed broach (Medin Barbed Broach, Vlachovice, Czech Republic). To prepare the root canals, the ProTaper system (Dentsply-Maillefer, Tulsa, OK, USA) and an endo motor E-Connect S electric motor (Eighteen Medical; Tds, China) at 300 rpm and torque 1 N cm according to the manufacturer’s instructions.were used. Crown- down technique was used, which included the application of a 3% sodium hypochlorite (NaOCl) irrigant (Egyptian Company for household detergents Clorox, Egypt) followed by 3 ml of 17% Ethylenediamine tetra acetic acid (EDTA) solution (AmritChem and Min. Ag, Mohali, India) and the canals were enlarged to the size of F4 file, maintaining a working length that is 1 mm shorter than the apex. finally, 5 ml of.

distilled water (Fipco, Egypt) followed by drying the canals using paper point size #35/0.04. After that, all canals were filled using the single cone obturation technique. The master cone 35/0.04 (Dentsply Maillefer, Ballaigues, Switzerland) was checked for tug back action in all samples. Finally, the specimens were attached to acrylic resin blocks by means of a parallelogram, which helped in mounting the samples in the universal testing machine during the push-out test [27]. 

### Intra-radicular post-space preparation

Post space preparation was carried out following the manufacturer’s guidelines. The gutta percha was removed using Gates-Glidden drills (Nordin, stainless steel, Switzerland). With a contra-angle handpiece ( NSK, GmbH, Eschborn, Germany) at 5000 rpm, post space was created with Glassix glass fiber special drills (Harald Nordin SA, Chailly/Montreux, and Switzerland) to a depth of 10 mm. The post space was then irrigated with a 3% NaOCl and EDTA solution to ensure complete removal of gutta percha and debris [28]. 

### Samples grouping

All samples were numbered from 1 and ascending, then were divided by the web site (www.random.org) into 4 equal divisions of samples according to the post-surface treatment as follows:

- *Group 1* (*n* = 6) control group: (silane only). A homogeneous coating silane coupling agent (Porcelain Primer/Bisco Inc, Schaumburg, IL, EUA). was spread over the post’s surface brush and then dried after 60 s by natural air.
- *Group 2* (*n* = 6): (30% hydrogen peroxide for 5 min + silane). The posts were immersed in a glass tube filled with 30% hydrogen peroxide for 5 min at room temperature. After etching with H2O2 ( El Nasr Pharmaceutical Chemicals Co., Egypt ), the posts were rinsed with distilled water for 2 min and air-dried for 10 s. Finally, a silane coupling agent was applied for 60 s.
- *Group 3* (*n* = 6): (sandblasting + silane). Sandblasting equipment (Cobra, Renfert GmbH, Hilzingen, Germany) was used to sandblast the posts with aluminum oxide particles (Korox 50, Bego, Bremen, Germany), that were 50 microns in size. The sandblast was applied perpendicularly to the surface of the post at a distance of 10 mm for 20 s and at a pressure of 2 bars. Finally, a silane coupling agent was applied.
- *Group 4* (*n* = 6): (Er: YAG laser + silane) [29]. The Er: YAG laser machine (Doctor Smile Erbium and Diode laser, Lambda Scientifica S.r.l., Vicenza, Italy) which is a pulsed laser system, emitting at a wavelength of 2,940 nm., was set with the parameters (150 mJ, 10 Hz, 1.5 W). Laser irradiation was done for 60 s, 100-µs pulse duration using a pulsed laser system. The optical tip, which had a diameter of 400 μm, was used at an incidence angle of 45 ° under water cooling (50% water – 50% air), 1-mm distant from the post surface from bottom to top. Finally, a silane coupling agent was applied.

### Scanning electron microscopy

**Fig. 2:**
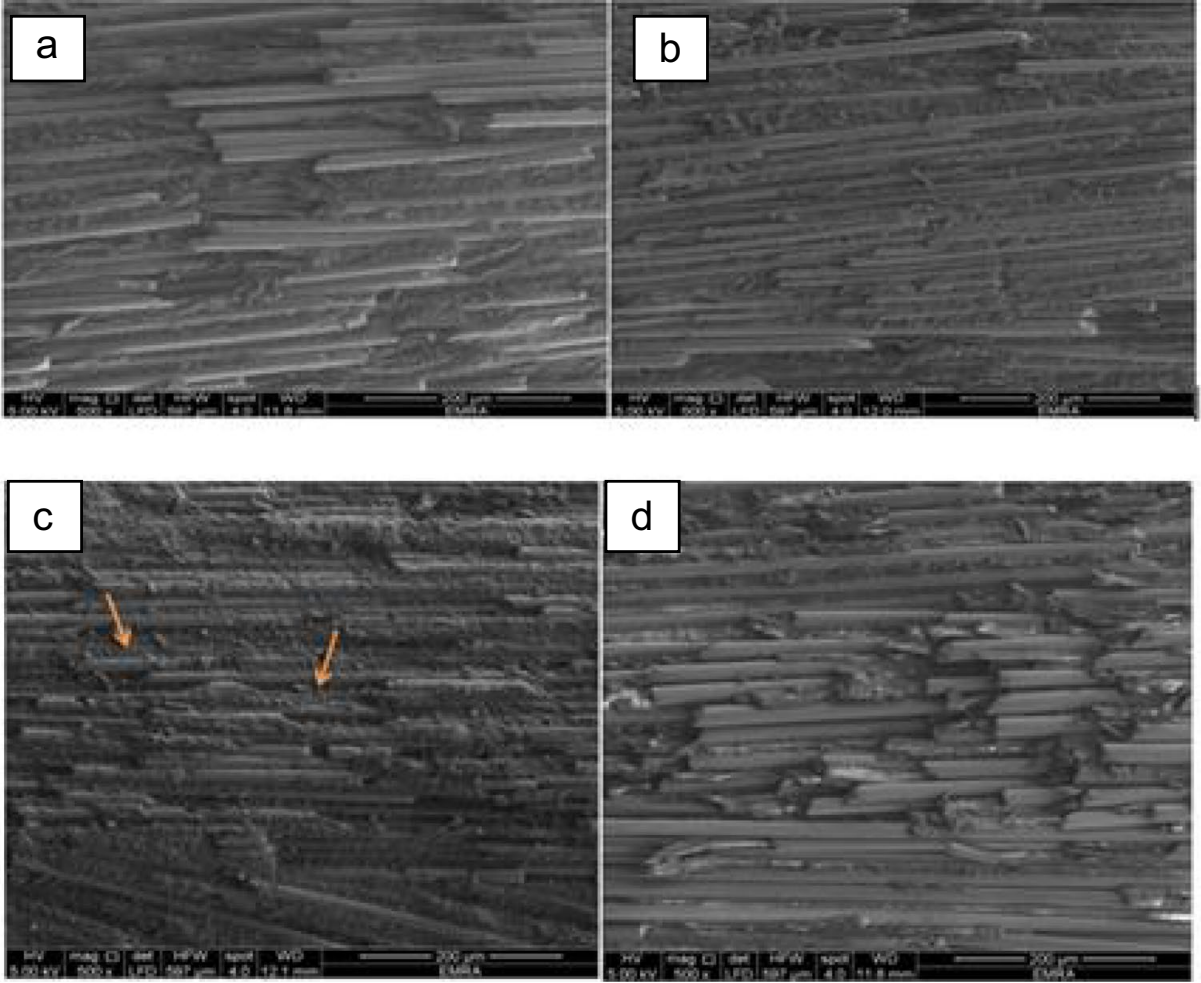
GFRC post under SEM image 500x for **a.** Control group, **b. **H2O2 group, **c.** sandblasted group, **d.** Er: YAG laser-irradiated group

To assess the impact of surface treatment on the post surface, a single specimen was randomly chosen from each group after surface treatment and before post cementation. The four samples were mounted on copper stubs with double-sided adhesive tape and were observed under a (SEM) 500x magnification as shown in Fig. 2.

### Fiber post cementation

GFP with diameters of 1.5 mm (Harald Nordin SA, Chailly/Monreux, Switzerland) were cemented using self-adhesive resin cement (SDI Limited, Victoria, Australia). Following a 24-hour storage period in distilled water at 37 °C, all specimens underwent thermocycling (2000 cycles at ± 5 °C and 55 °C, with a dwell time of 25 s in each water bath and a lag time of 10 s).

### Push-out test procedure

**Fig. 3:**
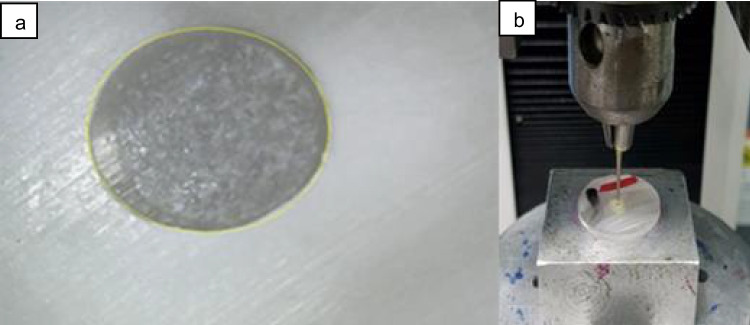
** a**. Slices photographed from apical and coronal surfaces using a stereomicroscope at magnification of 65x. **b**. Samples in universal testing machine

Each specimen was cut transversely perpendicular to the root’s long axis in order to get 1.5 mm ± 0.3 thick slices from the coronal, middle, and apical thirds. Every segment was recorded and photographed from the apical and coronal surfaces using a stereomicroscopy (SZ-PT; Olympus, Tokyo, Japan) at an initial amplification of 65x. as shown in Fig. 3a. In this study, the image analysis tool (Image J; NIH, Bethesda, MD) was used to build a ruler of given length. The Set Scale function was then used to compare the two rulers. After measuring the post’s diameter, the radius could be estimated. The next step was to subject the parts to compressive loads with a 1 mm/min crosshead speed on a universal testing machine (Model 3345; Instron Industrial Products, Norwood, MA, USA) as shown in Fig. 3b [30].

Analysis of the data was conducted at many stages. Firstly, the descriptive statistics for each group are obtained. A two-way analysis of variance (ANOVA) was conducted to identify the impact of each specific variable (surface treatment group and area). One-way analysis of variance (ANOVA) was conducted, followed by pair-wise Tukey’s post-hoc testing, to identify significant differences between major groups and radiation zones. Data visualizations were generated using Microsoft Excel. The statistical study was conducted using Aasistat 7.6 statistics software for Windows, developed by Campina Grande in Paraiba State, Brazil.

## Results

**Fig. 4:**
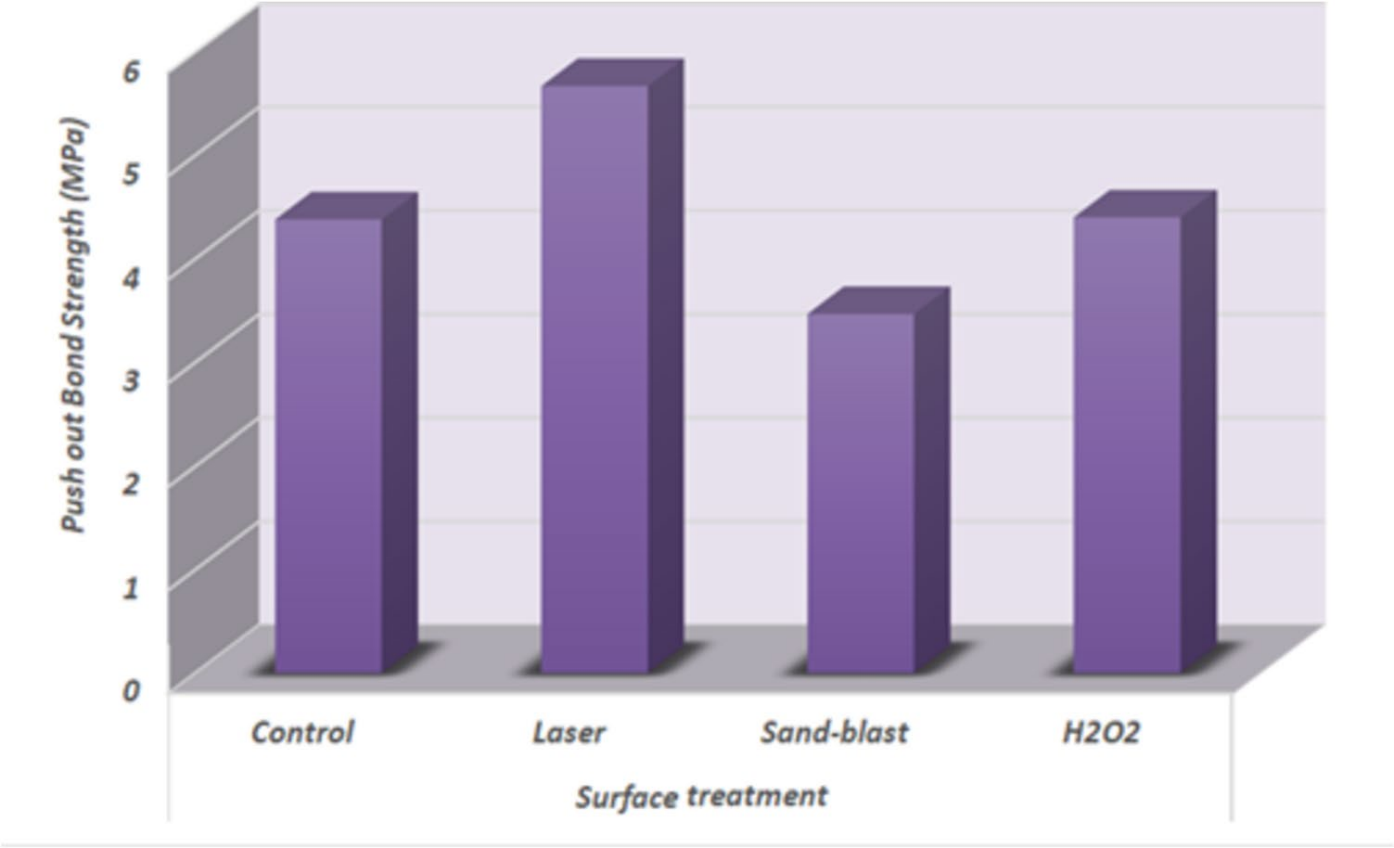
Column chart of push bond strength as a function of different surface treatments showing differences in the mean values between groups

The findings of the push-out bond strength tests for the various surface treatments were summarized in Table 1 and Fig. 4.

**Table 1:** Comparison of total push out bond strength results (Mean values ± SDs) as a function of surface treatment groups

Variables		Mean ± SDs	Statistics
*Surface treatment*	***Control***	**4.379534** ***±*** **1.64**^**ab**^	***P value***
	***Laser***	**5.668042** ***±*** **1.16**^**a**^	0.0013 *
	***Sand-blast***	**3.466738** ***±*** **0.98**^**b**^	
	***H***_***2***_ ***O***_***2***_	**4.400203** ***±*** **0.87**^**ab**^	

*S significant difference (*p* < 0.05), Mean push-out bond strengths (MPa), and standard deviations(SD) of different surface-treated glass fiber posts. Different superscript letters denote significant differences between groups

Regardless to radicular regions, totally it was found that that Laser treated group recorded the highest mean ± SD value of push out bond strength (5.668042 ± 1.16 MPa) followed by H2O2 treated group mean ± SD value (4.400203 ± 0.87 MPa) then Control non-treated group mean ± SD value (4.379534 ± 1.64 MPa) meanwhile the lowest mean ± SD value was recorded with Sand-blast treated group (3.466738 ± 0.98 MPa). The difference between groups was statistically significant as indicated by two-way ANOVA (F = 7.6, *P* = 0.0013 < 0.05). Pair-wise Tukey’s post-hoc test showed no-significant (*p* > 0.05) between (control and laser), (control and H2O2), (control and Sand-blast) and (Sand-blast and H2O2).

**Fig. 5:**
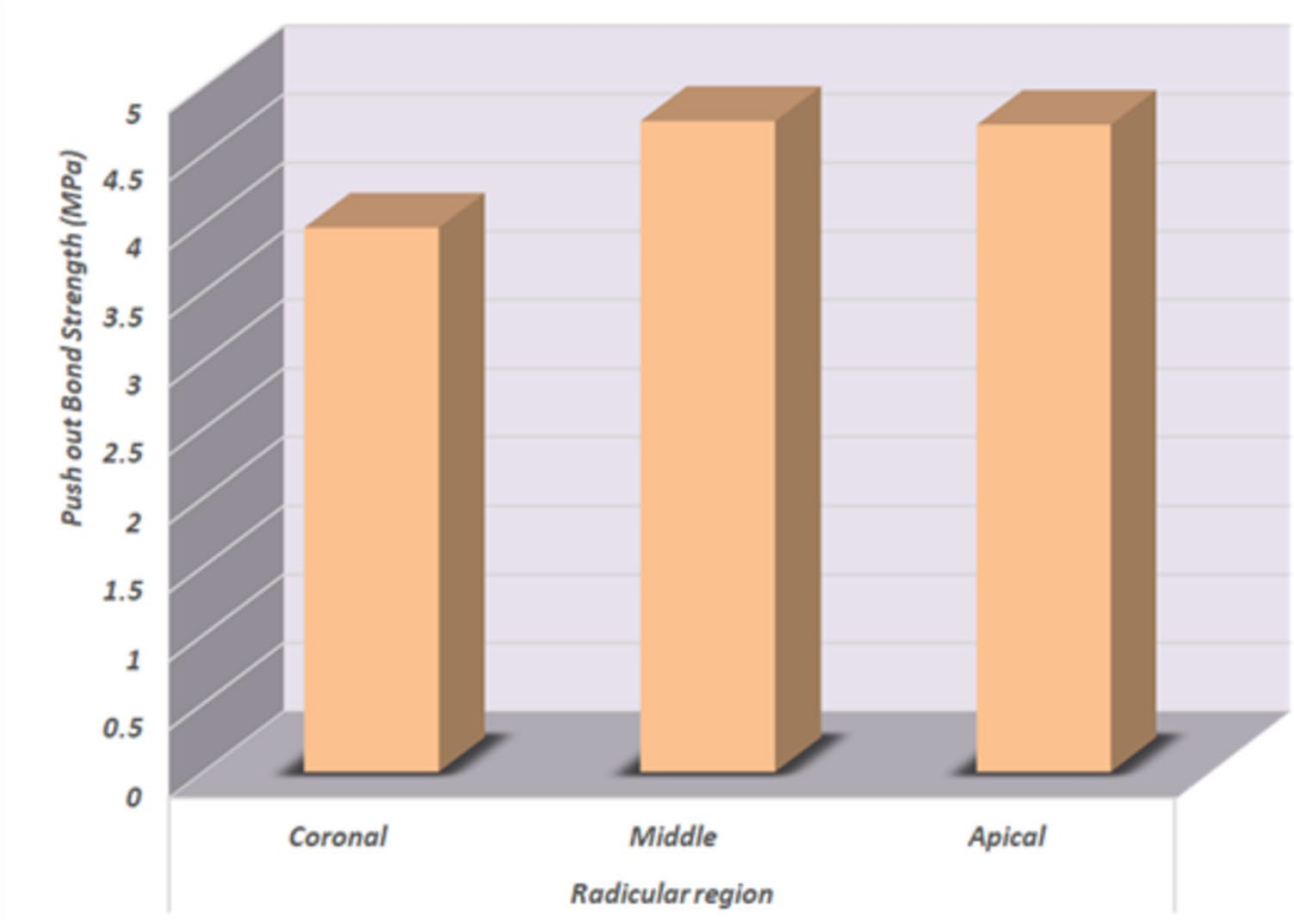
Column chart of push bond strength as a function of the radicular regions showing no differences in the mean values between groups

Regardless of surface treatment groups, it was found that the middle region had the greatest push-out bond strength, with a mean ± SD of 4.746851 ± 0.73 MPa, followed by the apical region mean ± SD value of (4.720187 ± 0.49 MPa), while the lowest mean ± SD value was recorded with the coronal region (3.968848 ± 1.25 MPa). Table 2 and Fig. 5, show that the two-way ANOVA (F = 2.6, *P* = 0.0879 > 0.05) showed that there was no statistically significant difference between the subgroups of the radicular region.

**Table 2:** Comparison of total push out bond strength results (Mean values ± SDs) as function of radicular region

Variables		Mean ± SDs	Statistics
*Radicular region*	***Coronal***	**3.968848 ± 1.25**^**a**^	***P value***
	***Middle***	**4.746851 ± 0.73**^**a**^	**0.0879 ns**
	***Apical***	**4.720187 ± 0.49** ^**a**^	

*ns non-significant (*p* > 0.05). Mean push-out bond strengths (MPa), and standard deviations (SD) of different radicular regions. Different subgroups were statistically non-significantly. Same superscript letters denote non-significant difference between sub-groups

## Discussion

Despite several comparative studies demonstrating the benefits of different surface pretreatment approaches for fiber posts, the literature lacks consensus regarding the most effective surface pretreatment approach for achieving optimal bonding [18, 31]. 

The results of our study showed that the groups treated with lasers, H2O2, and the control group had the strongest push-out bond strength in that order. The group treated with sandblasting, on the other hand, exhibited the weakest push-out bond strength. Irrigation protocol was done to enhance bond strength of GFP to dentin walls, as according to previous study [32], EDTA + laser-activated irrigation significantly reduces debris or smear layers from the root dentin wall.

Silane coupling agents have been suggested by several studies as a way to improve the fiber post’s adherence to resin cement [33–35]. Enhancing the formation of a covalent bond between the silane coupling agent, the resin cement, and the exposed glass fibers of the fiber post improves the surface wettability [36]. Researches indicate that silanization enhances the retention of glass fiber posts only when the post undergoes suitable surface pretreatment prior to silane application [9, 37]. 

The integrity of the bonding between silane and the GFP is diminished when the glass fibers are encased in a strongly cross-linked, non-reactive epoxy resin matrix [38]. 

The current investigation employed a 30% concentration of H2O2 for 5 min using the immersion technique, in accordance with recommendations from a prior study [39]. The hypothesis was that performing etching of the fiber post with H2O2 prior to applying silane would improve the adhesion between the resin cement and the glass fiber posts, leading to a higher push-out bond strength compared to the control group [40]. 

This phenomenon was observed due to the selective dissolving of the epoxy matrix by H2O2 through substrate oxidation while leaving the glass fibers intact and exposed for silanization. SEM images of H2O2 group Fig. 2b. have shown that dissolution of epoxy resin from GFP surfaces reveals the fibers and facilitates the formation of extra gaps for micro-mechanical retention of resin cements [41]. 

Recently, lasers have been employed for surface cleaning of materials, optimization of wettability, and increase of adhesion and stability of adhesive surfaces. Various laser types, such as Erbium yttrium scandium gallium garnet (Er, Cr: YSGG), Erbium-doped yttrium aluminum garnet (Er: YAG), and neodymium-doped yttrium aluminum garnet (Nd: YAG), are employed in dentistry with improved results [42]. 

In the irradiated area, water molecules and OH − groups absorb the Er: YAG laser, resulting in a rapid temperature increase. The heating characteristic of this process stimulates the evaporation of water molecules, resulting in increased pressure within the tissue and triggering micro explosions [43]. This procedure is termed ablation, resulting in morphological alterations in the hard tissue. For optimal outcomes with the Er: YAG laser and to enhance ablation, the presence of water molecules in the treatment area is crucial [44]. The current power level of 1.5 W for the Er: YAG laser was suggested in a previous study [19], that showed that all the surface glass fibers of the posts were safely exposed without any damage at this power, as according to previous study [45] laser in high power density cause changes in the structural characteristics of GFP and decrease the flexural strength and flexural modulus values. The application of Er: YAG laser post surface treatment significantly improved the push-out bond strength relative to other groups.

This behavior can be due to the action of the Er: YAG laser, which facilitates the ablation of the resin matrix on the surfaces of GFP, thus revealing the glass fibers. As a result, this technique results in the creation of a rough and irregular surface, featuring microretentive areas on the GFP surface [24]. The results of the SEM show that this hypothesis is true Fig. 2d. The ablation method selectively eliminates a thin layer of the epoxy resin matrix, therefore preserving the glass fibers intact. The surface displayed no residual debris inside the fibers, hence enhancing the micromechanical interlocking of resin cement to the post surface. This was in accordance with a study by Dikec et al. [46], Abohajar et al. [47], and Bitter et al. [48], who concluded that fiber post-surface treatment by Er: YAG laser increases its bond strength to resin cement and reduces adhesive failures, cement-dentin gap formation, and nanoleakage. While Mekky et al. [49]achieved a superior outcome with the Er-Cr: YSGG laser. This was in contradiction to Kurt et al. [50] who stated that the Er: YAG group exhibits lower bond strength compared to the sand-blasted group and Križnar et al. [51], who find that the Er: YAG group exhibits reduced bond strength relative to the untreated group. While Akin et al. [52] found that Sandblasting and Er: YAG laser-irradiation of the surface of the quartz fiber post before cementation is recommended for increasing retention. The differences in outcomes may be ascribed to the type of fiber post (quartz fiber post versus glass fiber post) and the specific setup parameters employed.

The sandblasting of the GFP surface increase its surface area, facilitating interaction between the glass fibers and the silane coupling agent [53]. The sandblasting process causes morphological and dimensional alterations that rely on pressure, duration, and particle size parameters [54]. This study involved the application of 50-micron Al2O3 powder at a pressure of 2 bar for 20 s with a nozzle distance of 10 mm. The choice of this particular particle size was determined by its capacity to induce surface alterations in the post without undergoing deformation [29]. Although sandblasting effectively roughens the surface of fiber posts to improve adhesion, it may also causes damage on the glass fibers and resin matrix, as seen in Fig. 2c, by causing disruption at the interface between the fibers [55]. This may explain the low results of the push-out bond strength, as the prolonged blasting procedure might be a contributing factor for this result, resulting in an excessive removal of the resinous matrix instead of just the outermost layer. As a result, it produced minor irregular surface roughness, which could hinder optimal wetting by the silane coupling agent and lead to the formation of voids between the resin matrix of the post and the silane interface. This result is consistent with the research carried out by Soares et al. [56]and Subramani et al. [57], which found that sandblasting pretreatment before silane application results in reduced bond strength compared to silane application alone. In contrast, previous research conducted by Sahafi et al. [58] and Tuncdemir et al. [59], found no significant difference between the group subjected to sand blasting and the control group that did not receive any treatment. This finding contradicts the results of Kelsey et al. [60] and Albashaireh [61]that sandblasting produced greater bond strength compared to other surface roughening techniques.

Fiber posts are commonly cemented to root canals using adhesive luting cements that are based on resin. The efficiency after bonding could be compromised by the time-consuming and technique-sensitive multistep bonding process [62]. So, this research used adhesive- based resin cement as it demineralizes and infiltrates the tooth substrate, resulting in micromechanical retention.

This study selected the push-out examination due to its ease of execution, reduced incidence of cohesive failure, and lower standard deviation. Push- out testing revealed an even greater distribution of stresses by finite element analysis; it has also been reported to exert shear stress parallel to the GFP and resin cement interface, analogous to clinical conditions [63]. 

Our investigation revealed that, irrespective of surface treatment groups, the middle region exhibited the maximum push-out bond strength, followed by the apical region, whereas the coronal region had the lowest value. Despite the statistical insignificance of the bond strength variations among the radicular regions, the elevated values in the middle and apical regions may be attributed to the enhanced adaptation of the post to the root canal walls in these areas, as well as the reduced diameter of the post in the apical third, given that the morphology of the root canal closely resembled the shape, diameter, and taper of the posts [64, 65].

Moreover, according to Goracci et al. [66], the root canal morphology leads to an increase in cement thickness in the cervical region, which might have a detrimental impact on the retentive bond strength of the cemented fiber posts. Elnaghy et al. [67]performed more research and came to conflicting conclusions, with self-adhesive resin cements showing a decrease in bond strength values towards the apical third.

According to the results of our study, the hypothesis was partially rejected as in comparison to the silanized group, the sandblasting group produced a lower value for the bonding capacity of fiber post to resin cement, but the Er: YAG and hydrogen peroxide groups only showed higher values.

Regarding the limitations of this study, although thermocycling was used as an aging factor simulation, the use of thermomechanical cycling procedures could mimic the clinical situation more precisely, and also our study did not study the failure pattern of the bonding surface by SEM. The effect of various laser surface treatment characteristics on the adhesion of fiber post and resin cement should be investigated further.

## Conclusion

Within the limitations of the current study, the following can be concluded:

1. The utilizing the Er: YAG laser achieves remarkable surface enhancement applied to glass fiber posts, successfully enhancing their ability to adhere to resin cement.
2. Sandblasting decreases fiber post-retention to resin cement.
3. The hydrogen peroxide and the control groups give similar bond strength.

## Data Availability

No datasets were generated or analysed during the current study.
